# Matrix-preserving partial nail resection guided by a spike-like proximal nail plate for ingrown toenails: A 2-center retrospective case series

**DOI:** 10.1016/j.jdin.2026.09.007

**Published:** 2026-10-01

**Authors:** Yuto Yamamura, Kazuyasu Fujii, Maiko Kato, Sho Onishi, Atsushi Otsuka

**Affiliations:** a Department of Dermatology, Kindai University Hospital, Sakai, Osaka, Japan; b Division of Dermatology, National Hospital Organization Osaka Minami Medical Center, Kawachinagano, Osaka, Japan

**Keywords:** ingrown toenail, matrix-preserving surgery, mechanical conflict, nail matrix, partial nail resection, recurrence, retrospective case series, spike-like proximal nail plate

*To the Editor:* Partial nail resection for ingrown toenails is commonly combined with matrix ablation to reduce recurrence; however, matrix ablation irreversibly narrows the nail, and the certainty of evidence supporting its additional benefit remains limited.^1^, ^2^ Conversely, low recurrence rates have also been reported after matrix-preserving soft-tissue procedures,^3^ raising the possibility that recurrence depends not only on persistence of the matrix but also on unresolved mechanical conflict between the nail edge and lateral nail fold (Fig 1).

**Fig 1:**
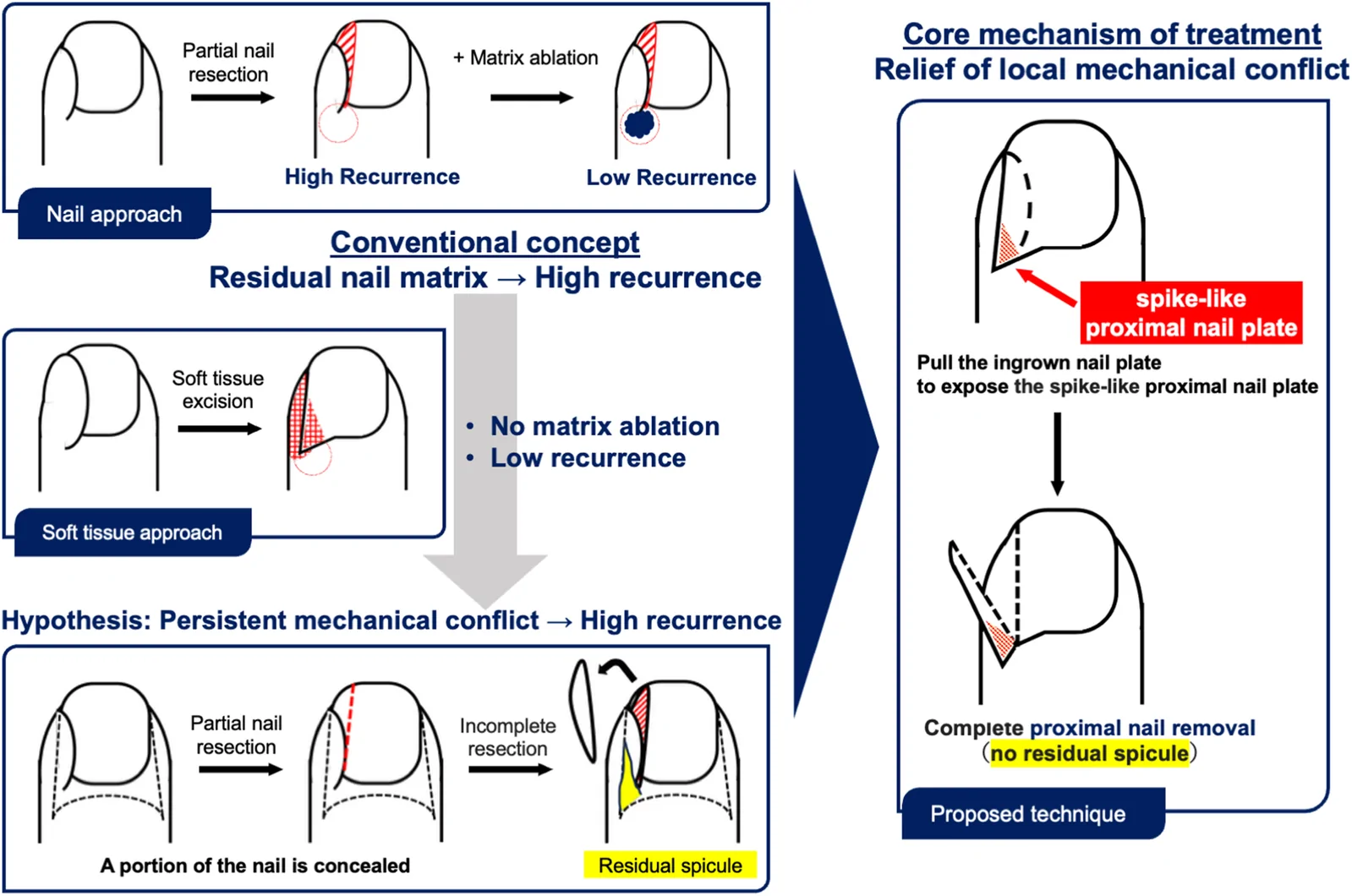
Ingrown toenail: mechanical-conflict framework and spike-like proximal nail plate–guided resection. Matrix ablation reduces recurrence after partial nail resection, whereas matrix-preserving soft-tissue procedures can also achieve low recurrence. These observations suggest that recurrence may depend not only on persistence of the nail matrix but also on unresolved mechanical conflict. The present technique directly exposes and removes the concealed proximal nail component while preserving the matrix.

During matrix-preserving partial nail resection, the proximal lateral nail plate is concealed beneath the nail folds and may be incompletely removed when resection is performed mainly from the distal side.^4^ We termed this concealed proximal extension the spike-like proximal nail plate (SLPNP) and developed a matrix-preserving technique in which it is directly exposed and excised to its proximal base (Fig 2).

**Fig 2:**
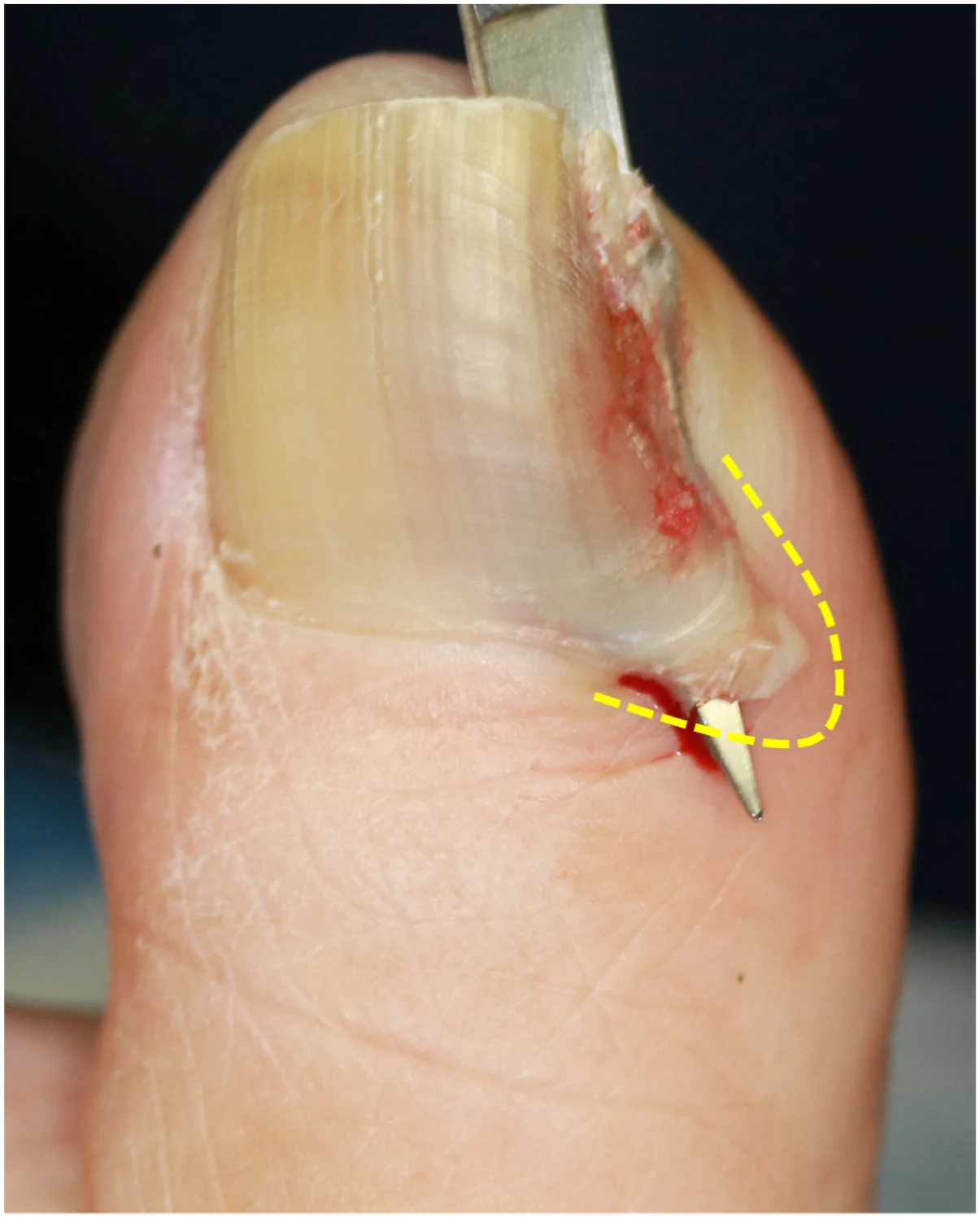
Ingrown toenail: direct visualization of the spike-like proximal nail plate. Proximal traction of the lateral nail plate exposes the concealed spike-like proximal extension beneath the nail fold, allowing direct visualization and excision to its proximal base without matrix ablation. The *yellow dashed line* indicates the spike-like proximal nail plate.

We retrospectively reviewed 20 patients with 30 nail plate–dominant ingrown great toenails treated at 2 institutions between April 2024 and September 2025. After proximal traction and release of soft-tissue attachments, the SLPNP was directly visualized and excised to its base without matrix ablation. The primary outcome was 6-month recurrence; pain and major complications were also assessed. The Institutional Review Board of Kindai University Faculty of Medicine granted centralized approval covering both institutions (R07-161); written informed consent for study participation was waived, with an opt-out opportunity. Detailed methods, cohort data (Supplementary Table I, available via Mendeley at https://data.mendeley.com/datasets/t89ntpp9ff/1), and operative and follow-up photographs (Supplementary Figs 1 and 2, available via Mendeley at https://data.mendeley.com/datasets/t89ntpp9ff/1) are provided in the Supplemental Material, available via Mendeley at https://data.mendeley.com/datasets/t89ntpp9ff/1.

No recurrence occurred at 6 months (0/30 toes; 0/20 patients; exact patient-level 95% confidence interval, 0% to 16.8%). Median follow-up was 9.9 months (range, 6.0-24.1), with 10 of 30 toes followed for at least 12 months and no recurrence at the last observation. Median pain on an 11-point numeric rating scale decreased from 6 preoperatively to 0 at 1 week and was 0 in all patients from 1 month onward. No major postoperative complications requiring treatment occurred.

These findings suggest that complete removal of the concealed proximal nail component may achieve favorable short-term outcomes while preserving the matrix in selected cases. The SLPNP is not a distinct anatomic structure but a practical intraoperative landmark that provides a visible end point for complete proximal nail removal. This approach prioritizes the release of mechanical conflict while avoiding matrix destruction and extensive soft-tissue excision.

Low recurrence after matrix ablation has generally been attributed to the suppression of subsequent nail growth.^1^, ^2^ However, access to the relevant matrix also requires removal of the corresponding proximal lateral nail plate.^5^ Thus, the reported benefit of matrix ablation may partly reflect more complete removal of the concealed proximal nail component and more complete mechanical-conflict release.

This small, selected, uncontrolled retrospective series cannot establish long-term recurrence control or equivalence to matrix ablation. The proposed mechanistic explanation remains untested. The SLPNP provides a visible operative end point for prospective comparison of standardized proximal nail removal with and without matrix ablation to assess the independent recurrence-preventive effect of matrix ablation.

### Declaration of generative AI and AI-assisted technologies in the writing process

The authors used ChatGPT (OpenAI) for English-language editing. The authors reviewed and revised all suggested changes and took full responsibility for the final content.

## Conflicts of interest

None disclosed.
